# Training Family Medicine Residents in Effective Communication Skills While Utilizing Promotoras as Standardized Patients in OSCEs: A Health Literacy Curriculum

**DOI:** 10.1155/2015/129187

**Published:** 2015-09-29

**Authors:** Patti Pagels, Tiffany Kindratt, Danielle Arnold, Jeffrey Brandt, Grant Woodfin, Nora Gimpel

**Affiliations:** ^1^Department of Family and Community Medicine, University of Texas Southwestern Medical Center, 5920 Forest Park Road, Dallas, TX 75390-9165, USA; ^2^Department of Physician Assistant Studies, University of Texas Southwestern School of Health Professions, 6011 Harry Hines Boulevard, Suite V4.114, Dallas, TX 75390-9090, USA; ^3^Pediatric Residency Program, University of Colorado School of Medicine, Children's Hospital Colorado, 13123 E. 16th Avenue, B-158, Aurora, CO 80045, USA

## Abstract

*Introduction.* Future health care providers need to be trained in the knowledge and skills to effectively communicate with their patients with limited health literacy. The purpose of this study is to develop and evaluate a curriculum designed to increase residents' health literacy knowledge, improve communication skills, and work with an interpreter. *Materials and Methods.* Family Medicine residents (*N* = 25) participated in a health literacy training which included didactic lectures and an objective structured clinical examination (OSCE). Community promotoras acted as standardized patients and evaluated the residents' ability to measure their patients' health literacy, communicate effectively using the teach-back and Ask Me 3 methods, and appropriately use an interpreter. Pre- and postknowledge, attitudes, and postdidactic feedback were obtained. We compared OSCE scores from the group that received training (didactic group) and previous graduates. Residents reported the skills they used in practice three months later. *Results.* Family Medicine residents showed an increase in health literacy knowledge (*p* = 0.001) and scored in the adequately to expertly performed range in the OSCE. Residents reported using the teach-back method (77.8%) and a translator more effectively (77.8%) three months later. *Conclusions.* Our innovative health literacy OSCE can be replicated for medical learners at all levels of training.

## 1. Introduction

Adequate health literacy plays a key role in effective communication between providers and patients. The 2003 National Assessment of Adult Literacy (NAAL) indicated that 36% of adults in the United States (US) had less than adequate levels of health literacy (basic or below basic) and several disparities existed among different demographic and socioeconomic groups [1]. Not only do patients with limited health literacy experience higher rates of hospitalization and increased risk of death, they also use preventive services less frequently, have limited understanding of health conditions, and may inappropriately take prescribed medications [2]. People with limited health literacy have difficulty with reading, comprehension, and basic numeracy required to appropriately take medicine and manage diseases [3]. These barriers may be exacerbated among our patients needing an interpreter when interacting with their providers [4, 5]. In order to reduce the health consequences among patients with limited health literacy, a comprehensive approach is needed that incorporates training health care providers with effective methods to overcome communication barriers and empower patients to become better managers of their health.

Curriculum should be designed to teach medical learners the knowledge and skills to determine health literacy levels of their patients and use techniques designed to overcome communication barriers caused by limited health literacy. Research has shown that medical residents often misjudge their patients' risk for limited health literacy [6]. Furthermore, residents frequently insert medical jargon or use medical terminology while talking with their patients [7]. Patients are often overwhelmed with too much information when they only want to know what to do when they get home. These skills are particularly important for Family Medicine residents for improving communication and encouraging treatment adherence and improved decision making [5, 8, 9]. Research is limited on curriculum designed to equip Family Medicine residents with the knowledge and skills to measure health literacy and communicate effectively with patients.

Several interventions have been developed to improve health outcomes among adults with limited health literacy [10]. However, few were designed to improve patient understanding and communication with providers. Ask Me 3 was developed by the Partnership for Clear Health Communication to improve communication between providers and patients. Ask Me 3 promotes patient understanding by encouraging patients to ask the following three questions during visits: (1) what is my main problem? (2) what do I need to do? and (3) why is it important for me to do this [11]? Research evaluating the effectiveness of Ask Me 3 techniques has produced mixed results. Interventions using Ask Me 3 among patients who already effectively communicate with providers did not change behavior [12, 13]. However, Mika and colleagues found that Ask Me 3 was effective at increasing communication between parents of pediatric patients and providers in a predominately Hispanic population [14].

A 2009 health literacy assessment (measured by the Newest Vital Sign) showed that 69% of our patients had less than adequate health literacy [15]. Over half of our patients (55%) completed the health literacy assessment in Spanish. Regardless of health literacy level, 47% reported that their main source of health information is their provider. After controlling for demographics, socioeconomic status, and health system related confounders, there were no differences in health information seeking behaviors between county and private clinic patients [15]. Given these findings, we developed a curriculum to increase residents' knowledge of health literacy, improve their communication skills with patients with limited health literacy, and work with an interpreter. Specific objectives of the* didactic and OSCE* curriculum* were linked and designed* to train residents to (1) administer and score the results of the Newest Vital Sign in English; (2) utilize patient-centered and clear health communication; (3) practice teach-back and Ask Me 3 during a mock-patient encounter; and (4) appropriately use an interpreter. In this paper, we provide an overview of the curriculum and effectiveness.

## 2. Materials and Methods

### 2.1. Design and Setting

We conducted a quasi-experimental study with Family Medicine (FM) residents at a county-supported indigent care clinic (*N* = 25) between February 2011 and February 2012. The residency includes 28 postgraduate year (PGY) residents on a three-year training (PGY1 *N* = 10; PGY2 *N* = 10; PGY3 *N* = 8). Our curriculum incorporates a unique education model that trains Family Medicine residents. Graduating residents are equipped with the knowledge, skills, and attitudes to meet the health needs of underserved communities [16].

### 2.2. Curriculum Development

Given the number of patients seen by residents that have low health literacy, we wanted to design a curriculum to specifically address this population. A brief letter describing the curriculum has been published previously [17]. The curriculum consisted of didactic learning and an objective structured clinical exam (OSCE) based on current literature in the field. The 90-minute didactic presentation consisted of short lectures, a video, and role-playing designed to teach FM residents the following four techniques: (1) employ patient-centered communication; (2) use clear health communication; (3) confirm patient understanding; and (4) provide reinforcement [2, 4]. The role-plays embedded into the didactic session were interactive in nature and consisted of triads where one resident acted as the patient, one resident acted as the provider, and one resident acted as an observer. These techniques were evaluated during the OSCE. OSCEs are used in medical education to evaluate clinical or communication skills [18]. OSCEs have a long history in medical education as either a formative or summative assessment of knowledge and skills. OSCEs use standardized patients to evaluate clinician performance and/or communication skills [18, 19]. We performed the one-hour OSCE with two groups of residents. One group completed the training 2 weeks after the didactic training and the other 4 weeks after the didactic training due to the complex scheduling with residency rotations. Residents completed four stations where they demonstrated the following: (1) administer and score the Newest Vital Sign; (2) use the Ask Me 3; (3) employ the teach-back method; and (4) work with an interpreter [11, 20]. Instead of standardized patients, we used six trained bilingual (English and Spanish) lay health promoters (promotoras) whose first language is Spanish. Promotoras are community health workers recruited from the communities they serve. Each receives Texas certification after 160 hours of training [21]. Promotoras “play an important role in promoting community-based health education and prevention in a manner that is culturally and linguistically appropriate, particularly in communities and for populations that have been historically underserved and uninsured” [22]. Our promotoras were given six hours of training on health literacy and scoring the OSCE. The lead author and a Texas certified trainer of promotoras developed and provided the training.

### 2.3. Data Collection

Two groups of FM residents were evaluated: (1) a didactic group (*N* = 18) who participated in a didactic presentation before the OSCE and (2) the control group (*N* = 7) that did not participate in the didactic before or after the OSCE. The didactic group included active residents and the control group contained previous third year residents near graduation. Outgoing PGY3 residents were chosen as a baseline because of their patient experience and the fact that they were not exposed to the health literacy didactic. The University of Texas Southwestern Medical Center's institutional review board exempted our study since it was considered quality improvement.

### 2.4. Measures

Our data was collected from several sources, including a pretest, posttest, and postdidactic evaluation, online follow-up survey, and OSCE score sheets. Current FM residents (didactic group) completed pre- and posttests to determine if the training had an immediate impact. The online follow-up survey was given three months after the OSCE to determine the residents' usage of the skills taught in training.

#### 2.4.1. Knowledge and Attitudes

Residents were asked eight multiple choice and fill-in-the-blank questions to determine health literacy knowledge. Questions ranged from definitions and national prevalence estimates to warning signs and consequences of limited health literacy. Residents were asked to rate their attitudes about health literacy and confidence in their ability to recognize and treat patients with limited health literacy on a 5-point Likert scale (1 = strongly disagree to 5 = strongly agree).

#### 2.4.2. Postdidactic Evaluation

After didactic training, residents were asked to evaluate the didactic on the same 5-point Likert scale. Residents evaluated whether or not the presentation improved their patient care, medical knowledge, practice-based learning, and/or improved interpersonal/communication skills. Qualitative feedback was given to determine strengths and ways of improving the training (see Table 3).

#### 2.4.3. OSCE

During the OSCE, lay health promoters evaluated the residents using a questionnaire with a three-point grading scale: (1) expertly performed, (2) adequately performed, and (3) did not perform. Each station was graded independently and had both general and specific questions. The grade sheet also allowed for comments from the grader regarding the residents' overall performance (see Table 1 for OSCE components).

#### 2.4.4. Three-Month Follow-Up

Three months after completion of the training, residents were asked to complete a 3-minute online survey (5 questions plus demographics) and give feedback. There were questions about their experience including how they have incorporated the skills they learned from the health literacy curriculum in their daily practice. Residents were asked if they have utilized or incorporated any elements from the health literacy training in their interactions with patients (yes or no) and if so, what elements were used (Newest Vital Sign, teach-back method, Ask Me 3, working with a translator, and/or other). They were also asked to provide feedback on what they found most helpful during the training, what was least helpful, and to describe their experiences of incorporating health literacy into their practice.

### 2.5. Data Analysis

Data analysis was performed using SPSS version 18.0. Means and standard deviations were used to report knowledge scores and attitudes. McNemar's test was obtained for each item of the knowledge assessment to determine improvement on specific questions measured. The Wilcoxon signed-rank test was used to determine whether total scores from the pre- and posttest and attitudes were significantly different. The Mann-Whitney *U* test was used to compare differences in OSCE scores from the intervention and control groups. Frequencies, percents, and qualitative quotations were compiled to report postintervention feedback.

## 3. Results

There were more female residents (56%) than males (44%) and most residents were in their first year (39%) compared to second (33%) and third years (29%) of training. Over half of the residents were Asian (56%) compared to 12% Hispanic, 12% White, and 8% Black. Race/ethnicity of 3 residents (12%) was unknown or not reported. Four residents had fluent Spanish-speaking skills. Two residents were native Spanish speakers.

### 3.1. Knowledge and Attitudes

Table 2 provides frequency counts and percentages of correct responses before and after didactic training. Results indicated a statistically significant difference for 1 out of 8 questions. Residents reported the greatest increase in knowledge of the definition of health literacy as “a constellation of skills, including the ability to perform basic reading and numerical tasks required to function in a health care environment” (*p* = 0.008) [1]. Overall, residents showed a significant increase in health literacy knowledge (*p* = 0.001). Residents also rated their attitudes towards health literacy as part of their residency training favorably both before and after the didactic. Although resident attitudes were high for all five questions before and after the didactic and there were no statistically significant differences, the greatest reported increase was in resident confidence at recognizing patients with low health literacy (*p* = 0.058).

### 3.2. OSCE

Table 2 also reports the means and standard deviations for both the control and didactic group for each OSCE station. The control group had higher scores on stations evaluating the teach-back method for asthma and on appropriately using a translator. Although not statistically significant, results showed the greatest difference in skill between the intervention and the control group on appropriately working with a translator (*p* = 0.075). The control and didactic group reported mean total scores in the adequately to expertly performed range for all four stations (OSCE1 = 4–8; OSCE2 = 11–22; OSCE3 = 8–16; OSCE4 = 8–16).

### 3.3. Postdidactic Evaluation

First and second year residents evaluated the didactic favorably (Table 3). Residents were most favorable about the presenters' knowledge (4.47) and felt that the stated objectives were met (4.47). Least favorable ratings were reported on the amount of time allowed for the training (4.27) and that the presentation provided them with medical knowledge of established and evolving biomedical, clinical, epidemiological, and social-behavioral sciences, as well as the application of this knowledge to patient care (3.93). Qualitative feedback indicated that some of the most important things learned during the training were the importance of health literacy and tools available to help patients. Some of the greatest strengths of the training were that it was interactive and included good patient scenarios and several available resources for patients and providers. With the support from supervisors, videos, and more classroom-based training residents reported that they would be able to implement what they learned in their practice. Potential improvements included more time and role-plays.

### 3.4. Three-Month Follow-Up

A majority of residents (87.5%) stated they believed the health literacy curriculum improved their patient care. A majority of residents (83%) stated that they have used the skills they learned in their training during their interactions with patients. The most utilized skills reported were the teach-back method (77.8%) and working with a translator (77.8%) compared to measuring patient health literacy with the NVS (27.8%) and teaching patients about Ask Me 3 (33.3%). Eighty-seven percent (87%) stated that the training aided their medical knowledge, and ninety-three percent (93%) stated the training improved their communication skills with their patients. When asked if they incorporated any components of the health literacy training into their practice and to describe their experience, one resident stated: “*Teach back method - A good number of times patients got it wrong meaning most of time in the past perhaps patient didn't fully understand instructions given. On teach back, most of the time they get it right after second explanation*.”

## 4. Discussion

The purpose of this study was to develop and evaluate a curriculum to train Family Medicine residents to effectively communicate with patients with limited health literacy. Our curriculum consisted of didactic training and a 4-station health literacy OSCE designed to teach residents to (1) administer, interpret, and document the results of the Newest Vital Sign; (2) utilize patient-centered, clear health communication and confirmation of understanding techniques; (3) practice the teach-back method and Ask Me 3 methods of communication; and (4) appropriately use an interpreter.

We found that residents showed a significant increase in health literacy knowledge after participating in a health literacy didactic and OSCE with standardized patients. A few educational interventions have been implemented to train physician residents on how to effectively communicate with low health literate patients [9, 23, 24]. Although some health literacy interventions have been developed to improve resident and medical student health literacy, there are no published studies evaluating a health literacy OSCE for Family Medicine physician residents. Kripalani and colleagues evaluated a health literacy training program for internal medicine residents that incorporated videotaped standardized patient encounters [24]. Standardized patients were trained for 3 hours on how to act as an adult with only some high school education and limited health literacy. Encounters took place in an OSCE format and residents viewed videotapes of their performance after the training [24]. Although this training included an OSCE, the researchers did not report a formal evaluation of the OSCE. Farrell evaluated a geriatric health literacy workshop designed to train medical students and residents in how to effectively communicate with older patients with low health literacy using an online portal. Although results indicated a significant improvement in self-assessment and communication skills among the medical students and residents, an evaluation of knowledge was not reported [23]. Our residents' predidactic attitudes towards the importance of identifying patients with limited health literacy remained positive. We found the greatest improvement in residents' confidence in recognizing a patient with low health literacy. Kripalani and colleagues found similar results with internal medicine residents five years after their initial study. Using an interactive workshop which emphasized recognition of limited health literacy, they found improved confidence in assessing adherence and medication counseling skills [9].

Our OSCE is innovative because it included a formal assessment of resident performance, it compared residents based on experience, and it utilized lay health promoter expertise. Although we did not find significant differences between the control and didactic group, control group residents had higher scores on the teach-back method for asthma and working with a translator stations. Since the control group residents were at the end of their residency training, the results may be due to experience and increased confidence. Previous studies of medical student and resident health literacy OSCEs have only reported program design and basic improvement of skills [24–26]. We compared OSCE scores among residents that attended the didactic to our control group that did not attend the didactic. Although not significantly different from each other, mean scores from both groups were high and fell into the adequately to expertly performed range. All residents evaluated the training favorably and the skills most often used after curriculum by residents were the “teach-back” method and working with an interpreter. Although Kripalani and colleagues did not report their OSCE results, residents rated the training favorably [24].

### 4.1. Strengths and Limitations

A great strength of this study was its reproducibility to other residency and health professional education environments. The active learning in the didactic was reinforced through the OSCE and it gave the residents an opportunity to practice with the promotoras and not just each other. Three months after the training, the knowledge gained from the intervention persisted. Residents reported using the teach-back method and improved their skills working with a translator. Furthermore, they also reported improvement in confidence and use of health literacy skills. Some limitations may have affected our results. Our study was conducted at 1 training site and included a small sample size. Future studies can be designed to include a larger number of residents and potential collaboration with other departments. There were logistical limitations with scheduling of residents and funding of promotoras as standardized patients. The promotoras were also only female. Two components of the training were the DASH diet and educating patients on how to use asthma inhalers. After the intervention, a few residents expressed a lack of confidence in counseling patients on the DASH diet and educating patients on how to use asthma inhalers.

## 5. Conclusions

Our health literacy OSCE addresses the urgent need to train medical learners and improve communication with low literacy patients. It provides a training model that can be used with residents and other students in the health professions. It meets Accreditation Council for Graduate Medical Education (ACGME) and Association of American Medical Colleges (AAMC) program requirements for patient communication skills. For example, although no specific core competencies on health literacy knowledge are currently required, Family Medicine residents are required to be able to communicate effectively with patients from different socioeconomic and cultural backgrounds [27]. More training is needed for existing and future health professionals. A one-time training is not sufficient to address the different subsets of limited health literacy. Tailored training is needed for population subsets (i.e., geriatric patients, parents, and cognitive impairments) and should be done earlier in medical education curriculum (3rd and 4th years or medical school). A recent survey of medical school deans reported that 72% of medical schools surveyed reported health literacy as part of their required curriculum. However, only 63 out of 133 schools (47.4%) responded to the survey and two schools declined to participate [28]. We believe that the foundation should be established in the latter years of medical school and residency should be a time of refinement of skills learned as a medical student not an introduction to our patients with low health literacy.

## Figures and Tables

**Table 1:** OSCE components.

OSCE station	Measures	Scoring		
		Did not perform (0 pts)	Adequately performed (1 pt)	Expertly performed (2 pts)
(1) Obtaining the Newest Vital Sign (NVS) *Score range: 0 to 8*	Explained the purpose of the NVS to the patient.	Examiner clearly did not perform this function.	Examiner explained the purpose of the Newest Vital Sign (NVS) and read questions to patient. Examiner did not accurately score the NVS and/or did not determine patient's health literacy.	Examiner explained the purpose of the NVS in a nonjudgmental way, read questions, and gave the patient time to provide an answer. Examiner accurately scored and determined the patient's level of health literacy.
	Read the questions on the NVS to the patient.			
	Scored the NVS according to instructions.			
	Used NVS score to determine patient health literacy.			

(2) Teach-back method for asthma *Score range: 0 to 22*	Sat while speaking with patient. Spoke slowly. Used words the patient could understand. Treated the patient with respect. Gave the patient the right amount of information for the time allowed. Provided clear instructions about how to use 2 inhalers. Asked the patient to repeat back, in a sensitive manner, what was taught about their 2 asthma inhalers. Was patient when the patient did not get some of the instructions correct the 1st time. Used additional methods to instruct the patient about the inhalers (drawings, demonstrations, etc.). Reviewed what the patient did not understand. Patient felt they could use these inhalers correctly after this education session.	Examiner clearly did not perform this function.	Examiner was relaxed and confident and seemed to care about the patient. The examiner gave information that was usually clear and easily understandable. Most of the words used by the examiner were easily understood. The patient felt comfortable repeating back what the examiner taught.	Examiner was relaxed and confident and cared about the patient. He/she sat down and gave the patient information about how to use the 2 inhalers in a way that was easy to understand. The patient did not feel overwhelmed. Examiner used more than just words to teach how to use the inhalers. The patient was able to “teach back” all information given correctly. The patient felt respected through the entire visit. The examiner never used words that the patient did not understand. The patient felt confident to go home and use these 2 inhalers without further instruction.

(3) Explain the DASH diet to hypertensive patient using Ask Me 3 *Score range: 0 to 16*	Explained that the main problem is high blood pressure.	Examiner clearly did not perform this function.	Examiner explained the DASH diet using words and terms the patient could understand. Examiner used at least one of the Ask Me 3 questions while explaining the DASH diet.	Examiner explained the DASH diet using nonmedical words and terms that the patient could understand. The examiner reviewed all Ask Me 3 questions with the patient.
	Used the word high blood pressure to describe main problem.			
	Described the DASH diet to help reduce blood pressure.			
	Gave the patient examples of what to eat in the DASH diet.			
	Explained why it is important to lower blood pressure.			
	Used nonmedical terms.			
	Used words/abbreviations and lay terms.			
	Defined Ask Me 3.			

(4) Working with an interpreter *Score range: 0 to 16*	Positioned interpreter behind or to the side.	Examiner clearly did not perform this function	Through most of the encounter, examiner faced the patient, spoke to the patient and not the translator, used the first person, employed plain language (not jargon or medical terms), and refrained from conversing with the interpreter.	Through the entire encounter, the examiner faced the patient, always made eye contact with the patient, always used the first person, did not have extra conversations with the interpreter, and used plain language.
	Introduced themselves to the patient through an interpreter.			
	Maintained eye contact with the patient.			
	Spoke to the patient and not to the interpreter.			
	Used the first person when talking to patient.			
	Refrained from carrying on side conversations with interpreter.			
	Used plain language.			
	Used brief statements and provided interpreter with time to relay information.			

**Table 2:** Health literacy knowledge^*∗*^, attitudes^§^, and OSCE station scores^*∗∗*^, *N* = 25.

Knowledge	Pretest Correct *N* (%)	Posttest Correct *N* (%)	*p* value
(1) How many Americans read at or below the 5th grade level according to NALS	5 (29.4)	4 (22.2)	0.625
(2) How many Americans have fair to low health literacy	5 (27.8)	6 (33.3)	1.000
(3) Definition of health literacy	6 (33.3)	14 (77.8)	0.008^±^
(4) Age groups likely to have low health literacy and worse health outcomes	14 (77.8)	15 (83.3)	1.000
(5) Communication styles among patients with limited health literacy	13 (72.2)	10 (55.6)	0.250
(6) Three questions that the Ask Me 3 Program comprises	2 (11.1)	17 (94.4)	0.000
(7) Definition of the “teach-back” method	12 (66.7)	16 (88.9)	0.125
(8) Health literacy assessment methods currently used.	13 (72.2)	17 (94.4)	0.125
Total score mean (SD)	**4.0 (1.41)**	**5.5 (1.50)**	**0.001** ^+^

Attitudes	Pretest Mean (SD)	Posttest Mean (SD)	

(1) Health literacy is a serious medical issue	4.50 (0.51)	4.33 (0.49)	0.180
(2) It is my responsibility, as a physician, to address my patient's health literacy	4.17 (0.51)	4.33 (0.49)	0.180
(3) I am confident I can recognize a patient with low health literacy	3.72 (0.67)	4.06 (0.56)	0.058
(4) I have the appropriate level of skills and training to help my patients with low health literacy	3.78 (0.65)	4.00 (0.61)	0.157
(5) Patients with low health literacy should be referred to a health educator or a social worker for health education	3.61 (0.98)	3.44 (1.29)	0.467

OSCE station scores	Control Mean (SD)	Didactic Mean (SD)	

(1) Obtaining the Newest Vital Sign (NVS)	5.0 (1.53)	5.8 (1.99)	0.459
(2) Teach-back method for asthma	17.4 (5.53)	17.1 (5.00)	0.480
(3) Explaining the DASH diet to a hypertensive patient using Ask Me 3	11.9 (3.24)	12.0 (2.61)	0.760
(4) Working with an interpreter	15.9 (0.39)	14.5 (2.72)	0.075

^*∗*^Total scores ranged from 0 to 8.

^+^Significant, *p* < 0.05, McNemar's test.

^±^Significant, *p* < 0.05, Wilcoxon signed-rank test.

^§^Five-point Likert Scale (1 = strongly disagree, 2 = disagree, 3 = neutral, 4 = agree, and 5 = strongly disagree); Wilcoxon signed rank test was used to test significance.

^*∗∗*^OSCE station 1 scores: 0 to 8; OSCE station 2 scores: 0 to 22; OSCE station 3 scores: 0 to 16; OSCE station 4 scores: 0 to 16; Mann-Whitney *U* test was used to test significance.

**Table 3:** Postdidactic evaluation^*∗*^, *N* = 16.

Questions	Mean (SD)
(1) This presentation met my needs	4.33 (0.49)
(2) The presenters were knowledgeable	4.47 (0.64)
(3) The techniques used were effective to teach the subject matter	4.33 (0.72)
(4) The stated objectives were met	4.47 (0.52)
(5) The amount of time allowed for material was appropriate	4.27 (0.80)
(6) The presentation enhanced my ability to provide care that is patient centered, compassionate, appropriate, and effective for the treatment of health problems and promotion of health	4.40 (0.51)
(7) This presentation provided me with medical knowledge of established and evolving biomedical, clinical, epidemiological, and social-behavioral sciences, as well as the application of this knowledge to patient care	3.93 (1.03)
(8) This presentation assisted me in developing skills and habits that will help me to self-evaluate and improve my care of patients	4.33 (0.49)
(9) This presentation assisted me in the development of interpersonal and communication skills that result in the effective exchange of information and collaboration with patients, their families, and health professionals	4.33 (0.62)
(10) Overall rating of this session	**4.21 (0.58)**

(11) What are the three most important things you learned during this training?	
* “Importance of health literacy” *	
* “Tools available to help patients (i.e. Ask Me 3, teach-back)”*	
* “Written instructions may not help”*	
(12) What are the three greatest strengths of this training?	
* “Interactive”*	
* “Good patient scenarios”*	
* “Examples of availability of resources”*	
(13) What additional assistance or resources, if any, will you need to be able to implement what you have learned at this training?	
* “Supervisor support”*	
* “Videos”*	
* “Classroom based”*	
(14) If you were given the task of revising, adjusting, or redesigning this training, what would you change?	
* “More role play”*	
* “Need more time”*	
* “Nothing”*	

^*∗*^Five-point Likert Scale (1 = strongly disagree, 2 = disagree, 3 = neutral, 4 = agree, and 5 = strongly disagree).

## References

[1] Kutner M., Greenberg E., Jin Y., Paulsen C. (2006). *The Health Literacy of America’s Adults: Results From the 2003 National Assessment of Adult Literacy*.

[2] Sudore L. R., Schillinger D. (2009). Interventions to improve care for patients with limited health literacy. *Journal of Clinical Outcomes Management*.

[3] Ryan J. G., Leguen F., Weiss B. D. (2008). Will patients agree to have their literacy skills assessed in clinical practice?. *Health Education Research*.

[4] Williams M. V., Davis T., Parker R. M., Weiss B. D. (2002). The role of health literacy in patient-physician communication. *Family Medicine*.

[5] Safeer R. S., Keenan J. (2005). Health literacy: the gap between physicians and patients. *American Family Physician*.

[6] Bass P. F., Wilson J. F., Griffith C. H., Barnett D. R. (2002). Residents’ ability to identify patients with poor literacy skills. *Academic Medicine*.

[7] Powers B. J., Trinh J. V., Bosworth H. B. (2010). Can this patient read and understand written health information?. *The Journal of the American Medical Association*.

[8] Farrell M. H., Kuruvilla P., Eskra K. L., Christopher S. A., Brienza R. S. (2009). A method to quantify and compare clinicians’ assessments of patient understanding during counseling of standardized patients. *Patient Education and Counseling*.

[9] Kripalani S., Osborn C. Y., Vaccarino V., Jacobson T. A. (2011). Development and evaluation of a medication counseling workshop for physicians: can we improve on ‘take two pills and call me in the morning’?. *Medical Education Online*.

[10] Berkman N. D., Sheridan S. L., Donahue K. E. (2011). Health literacy interventions and outcomes: an updated systematic review. *Evidence Reports/Technology Assessments*.

[11] National Patient Safety Foundatio (1997). *Ask Me 3*.

[12] Galliher J. M., Post D. M., Weiss B. D. (2010). Patients’ question-asking behavior during primary care visits: a report from the AAFP national research network. *Annals of Family Medicine*.

[13] Miller M. J., Abrams M. A., McClintock B. (2008). Promoting health communication between the community-dwelling well-elderly and pharmacists: the Ask Me 3 program. *Journal of the American Pharmacists Association*.

[14] Mika V. S., Wood P. R., Weiss B. D., Treviño L. (2007). Ask Me 3: improving communication in a hispanic pediatric outpatient practice. *American Journal of Health Behavior*.

[15] Gutierrez N., Kindratt T. B., Pagels P., Foster B., Gimpel N. E. (2014). Health literacy, health information seeking behaviors and internet use among patients attending a private and public clinic in the same geographic area. *Journal of Community Health*.

[16] Shakil A., Gimpel N., Ohagi J., Dobbie A. (2009). A community action research experience (CARE) program for family medicine residents. *Family Medicine*.

[17] Pagels P., Kindratt T., Arnold D., Brandt J., Woodfin G., Gimpel N. (2013). Health literacy objective structured clinical exam for family medicine residents. *Medical Teacher*.

[18] Turner J. L., Dankoski M. E. (2008). Objective structured clinical exams: a critical review. *Family Medicine*.

[19] Harden McG. R., Stevenson M., Wilson Downie W., Wilson G. M. (1975). Assessment of clinical competence using objective structured examination. *British Medical Journal*.

[20] Weiss B. D., Mays M. Z., Martz W. (2005). Quick assessment of literacy in primary care: the newest vital sign. *Annals of Family Medicine*.

[21] Nichols D. C., Berrios C., Samar H. (2005). Texas’ community health workforce: from state health promotion policy to community-level practice. *Preventing Chronic Disease*.

[22] US Department of Health and Human Services (2012). *HHS Promotores de Salud Initiative*.

[23] Farrell T. W. (2011). Review of a geriatric health literacy workshop for medical students and residents. *Journal of the American Geriatrics Society*.

[24] Kripalani S., Jacobson K. L., Brown S., Manning K., Rask K. J., Jacobson T. A. (2006). Development and implementation of a health literacy training program for medical residents. *Medical Education Online*.

[25] Harper W., Cook S., Makoul G. (2007). Teaching medical students about health literacy: 2 Chicago initiatives. *American Journal of Health Behavior*.

[26] Manning K. D., Kripalani S. (2007). The use of standardized patients to teach low-literacy communication skills. *American Journal of Health Behavior*.

[27] Accreditation Council for Graduate Medical Education (2007). *ACGME Program Requirements for Graduate Medical Education in Family Medicine*.

[28] Coleman C. A., Appy S. (2012). Health literacy teaching in US medical schools, 2010. *Family Medicine*.

